# Redefining “abandoned” agricultural land in the context of reforestation

**DOI:** 10.3389/ffgc.2022.933887

**Published:** 2022-08-21

**Authors:** Karen D. Holl, Mark S. Ashton, Jacob J. Bukoski, Katherine A. Culbertson, Sara R. Curran, Thomas B. Harris, Matthew D. Potts, Yesenia L. Valverde, Jeffrey R. Vincent

**Affiliations:** 1 Department of Environmental Studies, University of California, Santa Cruz, Santa Cruz, CA, United States; 2 School of Forestry and Environmental Studies, Yale University, New Haven, CT, United States; 3 Moore Center for Science, Conservation International, Arlington, VA, United States; 4 Department of Environmental Science, Policy, and Management, University of California, Berkeley, Berkeley, CA, United States; 5 Center for Studies in Demography and Ecology, University of Washington, Seattle, WA, United States; 6 Nicholas School of the Environment, Duke University, Durham, NC, United States

**Keywords:** forest restoration, landholder, land use and land cover change, land tenure, remote sensing, tree planting

## Abstract

Global mapping efforts to date have relied on vague and oversimplified definitions of “abandoned” agricultural land which results in overestimates of the land area that is likely to support persistent increases in forest cover and associated carbon sequestration. We propose a new conceptualization of abandoned agricultural land that incorporates changes in landholding status over time into determining whether land should be considered as abandoned. In order to develop more realistic estimates of the amount of land available for reforestation, we recommend clearly defining how abandoned land is categorized, discerning who owns and has rights to use the land, and combining remotely sensed data with household/stakeholder surveys to understand landowner motivations for not cropping or grazing land.

## Introduction

As the world strives to reduce greenhouse gas emissions, natural forest regrowth and active tree planting are frequently proposed as mitigation pathways to sequester carbon through increases in above- and below-ground biomass and soil organic matter (Bastin et al., 2019; Cook-Patton et al., 2020). This reforestation process requires land. Although the portion of the Earth’s land surface used for agriculture (cropland and pastureland) continues to expand (Lambin and Meyfroidt, 2011), a growing literature (e.g., Yin et al., 2020; Crawford et al., 2022; Potapov et al., 2022) uses remotely sensed biophysical and land cover data to map the distribution of so-called “abandoned agricultural land” in high, middle, and low income countries. Goga et al. (2019) and Yin et al. (2020) estimate a great deal of abandoned agricultural land, whereas Crawford et al. (2022) and Potapov et al. (2022) indicate far less and even diminishing amounts. This estimation discrepancy may be due to differences in land cover measurement, but we argue that it also likely arises from the omission of information about landholding status and the complex landholder decision-making process.

These mapping efforts are constrained to variables that can be quantified at a large scale, and they typically define abandoned agricultural land as land that was previously used for agriculture (including grazing) but where intensive human management appears to have ceased (Supplementary Table 1; Li and Li, 2017; Goga et al., 2019; Gradinaru et al., 2020). Some definitions specify a minimum of 2–5 years of observations of non-use, whereas others specify observations of successional processes resulting in the natural establishment of forests or other secondary vegetation. Certain agricultural uses, such as recent fallow or pasture lands, make these types of observations difficult to detect in large-scale landcover maps.

Definitions of abandoned agricultural land based on remote observation of land cover changes over time often overlook the complexity of landholder decision-making and how landholder choices affect the cessation of agricultural use and potentially its later recurrence (Sloan, 2022). These definitions result in overestimation of the available land area for reforestation for climate mitigation and other purposes, as well as of the permanence of newly established forest cover (Estel et al., 2015; Schwartz et al., 2020; Piffer et al., 2022). We suspect that this is why Crawford et al. (2022) find such variation in abandoned land estimates. Moreover, these definitions and the semantics of the term “abandonment” imply a decline in land utility for agricultural livelihoods and a ceding of land rights to others. Using a definition that assumes away local residents’ landholding rights may inadvertently create social vulnerabilities in localities where there appear to be opportunities for reforestation. Without attention to landholding status and associated livelihoods, reforestation projects on abandoned agricultural land risk overstating their impact and sustainability.

We propose a new conceptualization of abandoned agricultural land that incorporates changes in landholding status over time into determining whether land should be considered as abandoned. While challenging to implement empirically, this conceptualization offers an improved approach to understanding the significant heterogeneity in land use changes—not simply land cover changes. Whereas land cover changes can be observed directly through remotely sensed imagery, land use changes cannot always be inferred from changes in land cover. For example, land that exists under “forest” cover could be used for selective logging, shade-grown agroforestry, or biodiversity conservation, each of which confers different environmental and socio-economic outcomes. These changes in land use ultimately derive from landholding status because landholding status affects landholders’ planning horizons. For example, medium- and long-term availability of abandoned land needed to sequester carbon and achieve other desired reforestation benefits depends on secure landholding that can only be known through incorporating information about landholding status.

We focus on land that was originally forested but then converted to an agricultural use that has currently ceased (i.e., the land is no longer being used to cultivate crops or graze livestock). We identify the decision points that determine whether this abandoned agricultural land follows pathways that lead to a persistent transition to forest, either natural forest cover or woody tree plantations *sensu* FAO (2018).

### Land abandonment and re-clearing of forest cover

A common assumption is that abandoned agricultural land is part of a “forest transition.” After a period of deforestation driven by agricultural expansion, economic development creates sufficient off-farm employment that the agricultural labor force shrinks, agricultural labor costs rise and profits fall, and some land shifts from agriculture to uses that are less labor-intensive, including secondary forest or tree plantations (Li and Li, 2017); often the land has low agricultural productivity. Forest transitions may also result from a rise in the price of wood rather than just a change in the relative return on labor (Rudel et al., 2005). This land is then commonly considered to be “abandoned” in terms of agricultural use, and it is assumed that forests will grow back on it through either natural regeneration or tree planting. This process has been documented in Europe, the eastern United States, Japan, Mexico, China, India, Brazil, and other regions worldwide (e.g., Rudel et al., 2005; Xiao et al., 2019; Gradinaru et al., 2020; Yin et al., 2020).

**FIGURE 1:**
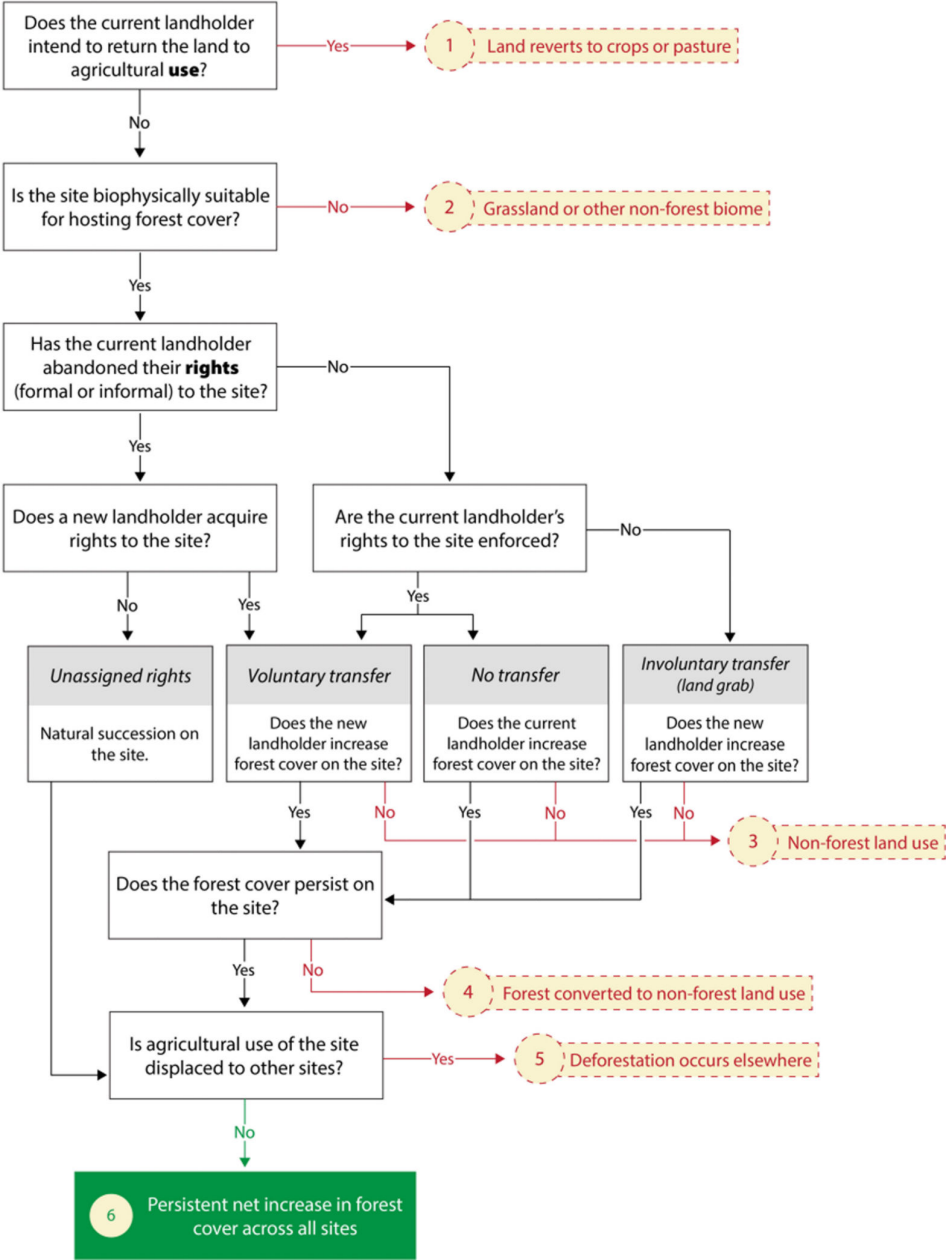
Flowchart of factors affecting whether temporary cessation of agriculture use of land results in a persistent net increase in forest cover. Red arrows indicate an exit from the possibility of a net increase in forest cover; green arrow indicates an arrival at a net increase in forest cover; black arrows indicate intermediate paths that do not lead directly to a change in forest cover. Numbered outcomes are referenced and explained in the text. Landholder includes both landowners and other people who use the land but lack clear title to the land.

In fact, abandoned agricultural land results from a series of land-use decisions that are influenced by complex biophysical, demographic, and socioeconomic processes (Benayas et al., 2007; Li and Li, 2017; Sloan, 2022). This means that land that is currently “abandoned” from agricultural use will not necessarily result in a persistent, net increase in forest cover (Figure 1). To begin, the current landholder might have ceased agricultural use only temporarily, leaving the land fallow for multiple years to allow soil nutrients to accumulate, pathogens to subside, or market conditions to become more favorable (Figure 1, outcome 1). Even if the current landholder’s intention is a permanent cessation of agriculture, the land might not be biophysically suitable for reforestation, either because it is highly degraded (e.g., salinization from agriculture, hydrologic alteration, soil compaction from grazing) or is in a biome where grassland or another low tree cover ecosystem is the naturally occurring vegetation (Figure 1, outcome 2).

Considerations of land tenure, namely the landholder’s and potentially others’ formal or informal rights to own or use the land and how these rights are enforced, add complexity when the current landholder “abandons” agricultural use of land that is suitable for forest regeneration. By ceasing agricultural use, the current landholder may or may not abandon their rights to the land. In some locations, ending agricultural use necessitates relinquishing land rights because land that is not cultivated or grazed is considered “idle” or unassigned, but this linkage of *use* abandonment and *rights* abandonment is not universal (Notess et al., 2021). If no new landholder acquires rights to the land, then natural succession will likely result in forest regeneration (Figure 1). However, if a new landholder acquires the rights to the land through voluntary sale or reassignment by a governmental or communal authority, then they, in turn, will decide whether to increase forest cover on it or revert to using it for agriculture or another non-forest purpose, such as urbanization (Figure 1, outcome 3). Forest regeneration is similarly not assured if the current landholder retains their rights to the land, as they too could decide to convert the land to a non-forest use (Sloan, 2022). In cases where the original or new landholder decides to increase forest cover on the site, then they also must decide whether to regenerate the forest using natural or artificial means, which entails considering a mix of ecological, silvicultural, and socioeconomic factors (Vincent et al., 2021).

A fourth possibility is that the current landholder’s rights are not enforced, in which case an involuntary land transfer occurs against the current landholder’s wishes: a “land grab.” For example, Ugandan villagers report that Norwegian investments in forest carbon plantations caused “forced relocations of agriculture, grazing, and other livelihood activities” (Richards and Lyons, 2016). A non-forest land use, typically some form of commercial agriculture, could also follow a land grab (Deininger and Byerlee, 2011).

Even if the landholder initially decides to establish forest cover on the land, the forest cover might not persist (Figure 1, outcome 4) for a host of reasons. Changes in agricultural prices and technologies, off-farm employment opportunities, wood scarcity, political instability, and government policies and subsidies can prompt landholders to reverse a decision to retain forest cover (reviewed in Yin et al., 2020; Sloan, 2022). For example, some of the extensive area of agricultural land abandoned in eastern Europe following the fall of the Soviet Union has been re-cultivated in the past decade, mostly due to increases in agricultural subsidies and commodity prices (Estel et al., 2015). Whereas it is commonly assumed that forest cover will increase as opportunities for non-agricultural employment increase and people migrate to cities, there are examples of reverse migration to rural areas when economic opportunities or personal situations change (Sloan, 2022). Climate change also decreases the likelihood of sustained forest cover increases in some locations due to changes in disturbance regimes and migration of both forest species and humans to more suitable climatic conditions.

Finally, forest cover changes must be evaluated at sufficient scale to determine whether there is a net increase over a region or the globe, given documented cases in which ceasing agriculture in one location has led to displacement (i.e., “leakage”) of agriculture to other locations (Meyfroidt et al., 2010; Yan, 2019), with any increase in forest cover in the first location being partially or completely offset by deforestation elsewhere (Figure 1, outcome 5). This displacement of deforestation can be challenging to quantify, as it may happen far from the study region given the ever-increasing globalization of the trade of forestry and agricultural products (Meyfroidt et al., 2010, 2022).

In sum, landholding status and land rights are complicated core factors affecting the net increase in persistent forest cover that ultimately results from abandoned agricultural land (Figure 1, outcome 6). Although agricultural and forestry policies, also mediate the use of abandoned agricultural land, we argue that landholding status and land rights are fundamental factors that cannot be ignored when implementing reforestation programs.

### Recommendations

Despite the challenge of mapping complex land-use decisions, there are important policy reasons to estimate how much land is potentially available for reforestation in the future. To that end we suggest the following. First, those undertaking these efforts should clearly state how they are defining “abandoned” land and other related terms (e.g., marginal, degraded^1^ ), including the temporal and spatial scale of those definitions. We recommend using more specific terms such as fallow (indicates that land may be part of a rotational cropping systems and likely will be returned to agriculture), agroforestry or silvopastoral systems (tree cover may be increasing while the land is still actively used for agricultural production), timber plantation (included in forest cover by the FAO but is harvested periodically), or secondary forest. We also advocate evaluating the persistence of these different land uses. These definitions are best defined regionally, given differences in production systems by locality and ecosystem. Likewise, it is important to more transparently acknowledge the complex suite of factors that affect forest transitions and reversals that are likely to bias estimates of land available for reforestation (Sloan, 2022).

Second, more attention should be paid to who owns and has rights to use the land when mapping global land uses and looking for investment opportunities for carbon or land purchase. In some regions of the world, land ownership maps exist that could be included as part of mapping efforts. In other regions, this can be challenging given issues of unclear land tenure (e.g., communal lands or nomadic pastoralism; Larson et al., 2013). In general, local stakeholders want tenure mapped and formally recognized, but tenure and land claims are often unclear or contested (Meyfroidt et al., 2022). Mapping tenure and resolving contested claims can hinder or complicate land-based projects and programs (Hoang et al., 2019), but is critical to their success. Further, absence of legally recognized tenure due to incomplete or partial mapping processes may invite land grabbing and induce conflict, particularly in common property systems (Dell’Angelo et al., 2017). Participatory mapping is a technique that can help illuminate and resolve potential conflicts but using this approach at broad geographic scales remains a challenge (Brown et al., 2017). Ultimately, it may not be possible to accurately map “abandoned” land in regions where land tenure is particularly contentious.

Third, a promising approach to developing more realistic and inclusive maps of abandoned agricultural land and potential forest cover is to combine remotely sensed data with household/stakeholder surveys to understand landowner motivations for not cropping lands. For example, Zhang et al. (2017) combined remotely sensed data of forest cover change with household surveys and focus groups to elucidate factors affecting increases in forest cover in northwest Yunnan Province, China. They found that forest cover increases were not greater on lands receiving payments from the Grain-for-Green program, in contrast to the reported net positive effect of the program on forest cover at the national level (Yan, 2019). Instead, the surveys and focus groups revealed that forest cover changes in the study area were influenced by a suite of factors that cannot be remotely sensed, including off-farm labor opportunities, changing energy sources, and tree crop planting for income (Zhang et al., 2017). We realize this combined approach is time consuming and necessarily will need to be done at a regional or local scale, but this is the scale at which most policy decisions are made, and the estimates of land area available for reforestation will be more realistic. Furthermore, attention to these details prior to major investments in reforestation will reduce the likelihood of significantly diminished, net zero, or net negative returns.

## Supplementary Material

Appendix - Data Sheet

## Data Availability

The original contributions presented in this study are included in the article/Supplementary material; further inquiries can be directed to the corresponding author.

## Footnotes

1 The term “abandoned” is often used interchangeably with marginal or degraded lands, equally vague terms that we do not discuss here.
