# Supplementary material for: Redefining “abandoned” agricultural land in the context of reforestation
Source: Front For Glob Change. Author manuscript; Available in PMC 2025 Oct 15. (PMC12520204; doi:10.3389/ffgc.2022.933887)
Supplement: Appendix - Data Sheet [file NIHMS2048035-supplement-Appendix_-_Data_Sheet.pdf]

Table S1. Range of ways abandoned agricultural land is defined in the literature

**Term**	**Definition**
Abandoned agricultural land (Kuemmerle et al., 2008)	includes both fallow land = "crops or managed grasslands (cut or intensively grazed) that had been replaced by unmanaged grasslands or successional shrubland" and reforestation = "natural or artificial reestablishment of forest cover in areas that had been converted to some other land use"
Abandoned farmland (Estel et al., 2015)	agricultural land that has not been used for at least five to six years
Abandoned farmland (Xiao et al., 2019)	"considered arable land barren for two or more years to be abandoned"
Agricultural land abandonment (Goga et al., 2019)	"in the most general interpretation by the available literature, is defined when the agriculture land is not managed (not sawn, not tilled, not even in the case of crop cultivation, not mown, or not grazed in the case of meadows), the formerly managed arable land and meadows are gradually replaced by unmanaged grass–herb formations and successive shrubs, or the agricultural land is not exploited for a minimum of five years"
Cropland abandonment (Yin et al., 2020)	"the land was not cultivated for at least five consecutive years"
Farmland abandonment (Pointereau et al., 2008)	"can be defined as the cessation of agricultural activities on a given surface of land and not taken by another activity (such as urbanisation or afforestation)"
Land abandonment (FAO, 2006)	"is a process, whereby human control over land (e.g. agriculture, forestry) is given up and the land is left to nature. After a number of years, depending on the ecological zones and climate, land can be considered as completely "abandoned", when either legal (e.g. forest law) or natural conditions (e.g. desertification, overgrowth with forest) render a restoration for agricultural use impossible or too costly"

References

Estel, S., Kuemmerle, T., Alcántara, C., Levers, C., Prishchepov, A., and Hostert, P. (2015). Mapping farmland abandonment and recultivation across Europe using MODIS NDVI time series. *Remote Sensing of Environment* 163**,** 312-325.

Fao (2006). *The role of agriculture and rural development in revitalizing abandoned/depopulated areas* Rome: Food and Agriculture Organization of the United Nations.

Goga, T., Feranec, J., Bucha, T., Rusnák, M., Sačkov, I., Barka, I., Kopecká, M., Papčo, J., Oťaheľ, J., Szatmári, D., Pazúr, R., Sedliak, M., Pajtík, J., and Vladovič, J. (2019). A review of the application of remote sensing data for abandoned agricultural land identification with focus on Central and Eastern Europe. *Remote Sensing* 11**,** 2759.

Kuemmerle, T., Hostert, P., Radeloff, V.C., Van Der Linden, S., Perzanowski, K., and Kruhlov, I. (2008). Cross-border comparison of post-socialist farmland abandonment in the Carpathians. *Ecosystems* 11**,** 614.

Pointereau, P., Coulon, F., Girard, P., Lambotte, M., Stuczynski, T., Sanchez Ortega, V., and Del Rio, A. (2008). *Analysis of farmland abandonment and the extent and location of agricultural areas that are actually abandoned or are in risk to be abandoned. European Commission Joint Research Centre.* Italy: European Commission.

Xiao, G., Zhu, X., Hou, C., and Xia, X. (2019). Extraction and analysis of abandoned farmland: A case study of Qingyun and Wudi counties in Shandong Province. *Journal of Geographical Sciences* 29**,** 581-597.

Yin, H., Brandão, A., Buchner, J., Helmers, D., Iuliano, B.G., Kimambo, N.E., Lewińska, K.E., Razenkova, E., Rizayeva, A., Rogova, N., Spawn, S.A., Xie, Y., and Radeloff, V.C. (2020). Monitoring cropland abandonment with Landsat time series. *Remote Sensing of Environment* 246**,** 111873.
